# Supplementary material for: Development of Stable Nano-Sized Transfersomes as a Rectal Colloid for Enhanced Delivery of Cannabidiol
Source: Pharmaceutics. 2022 Mar 25;14(4):703. doi: 10.3390/pharmaceutics14040703 (PMC9032849; doi:10.3390/pharmaceutics14040703)
Supplement: Supplementary file 1 [file pharmaceutics-14-00703-s001.zip › pharmaceutics-1639191-supplementary.pdf]

pharmaceutics

MDPI

Supplementary Materials: Development of Stable Nano-Sized Transfersomes as a Rectal Colloid for Enhanced Delivery of Cannabidiol

Thope Moqejwa, Thashree Marimuthu, Pierre P.D Kondiah and Yahya E. Choonara

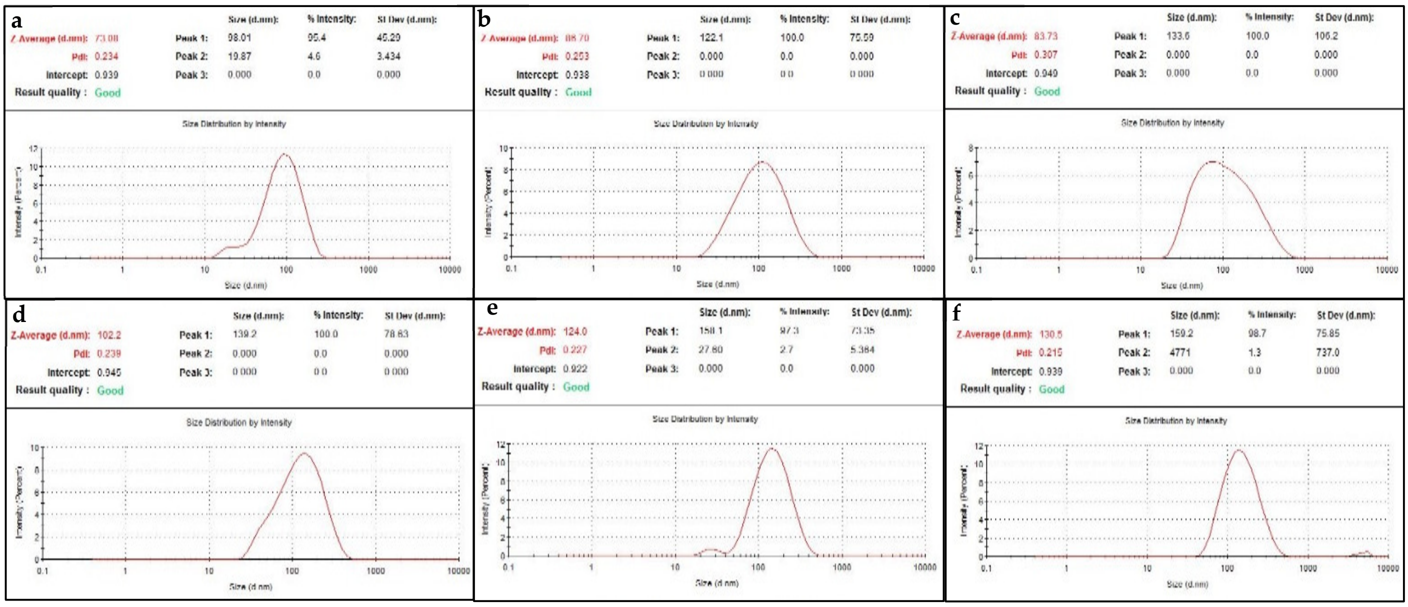

Figure S1. Size of non-lyophilised transfersomes colloid. **(a)** T1, (**b)** T2 (**c**) T3, (**d)** T4, (**e**) T5 and (**f**) T6.

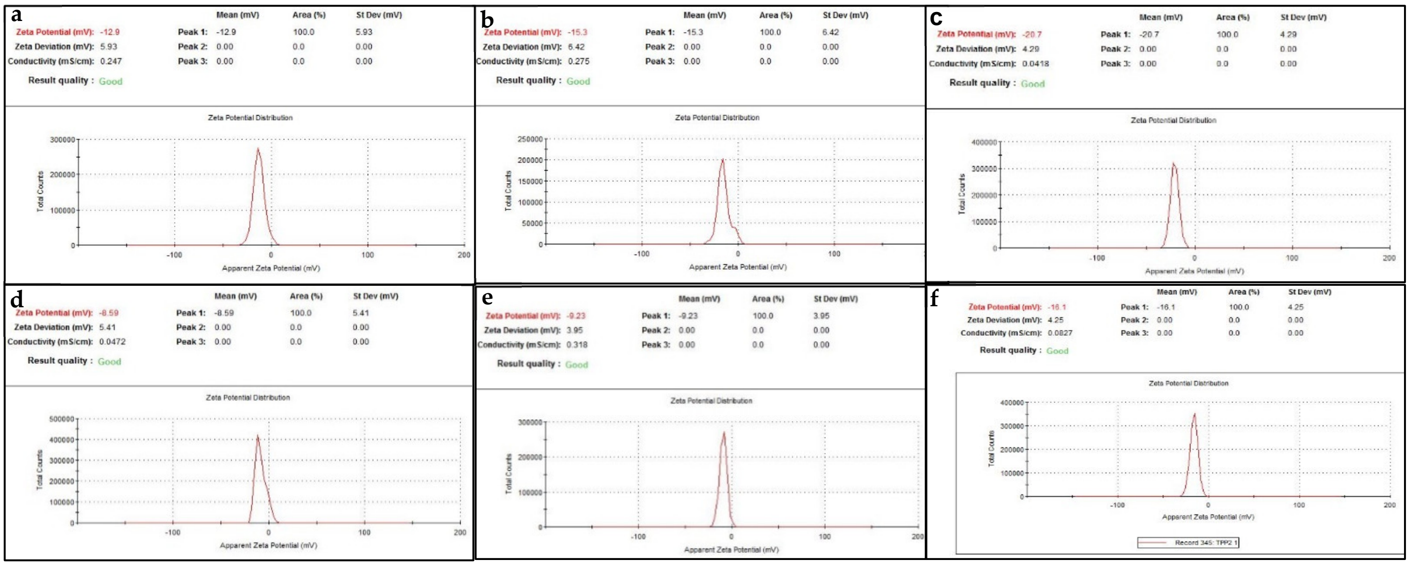

Figure S2. Zeta potential of non-lyophilized transfersomes colloid. **(a)** T1, (**b**) T2, (**c**) T3, (**d**) T4, (**e**) T5, and (**f**) T6

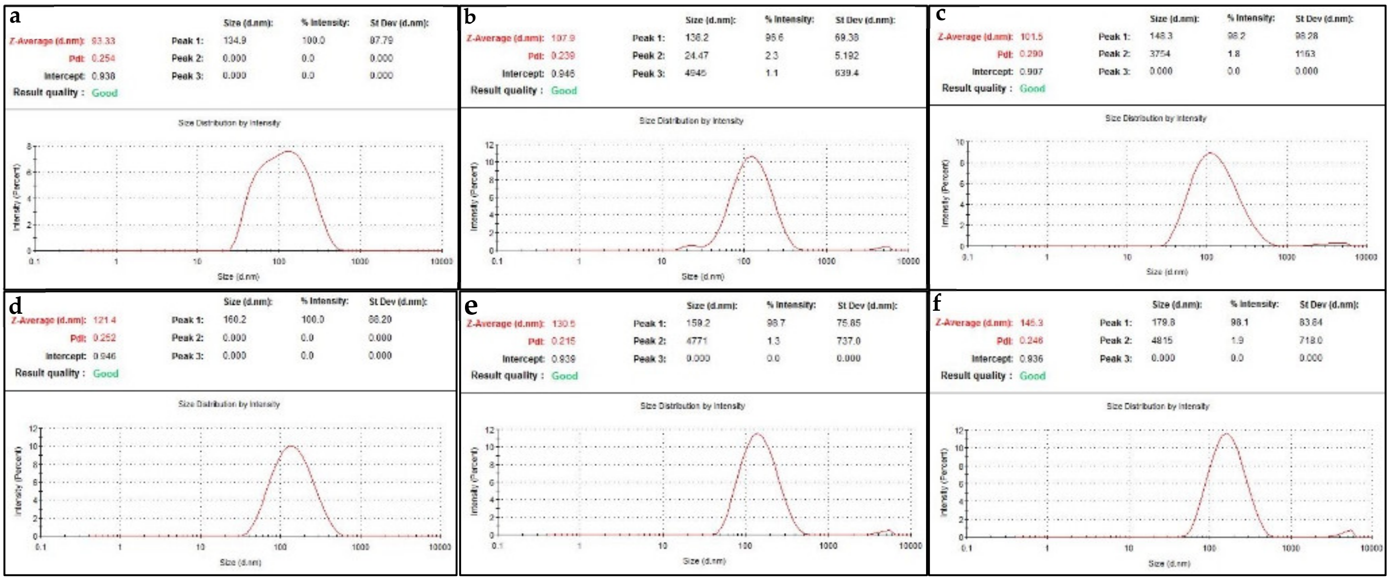

Figure S3. Size of lyophilized transfersomes colloid**. (a)** T1, (**b)** T2, (**c)** T3, (**d)** T4, (**e**) T5, and (**f)** T6.

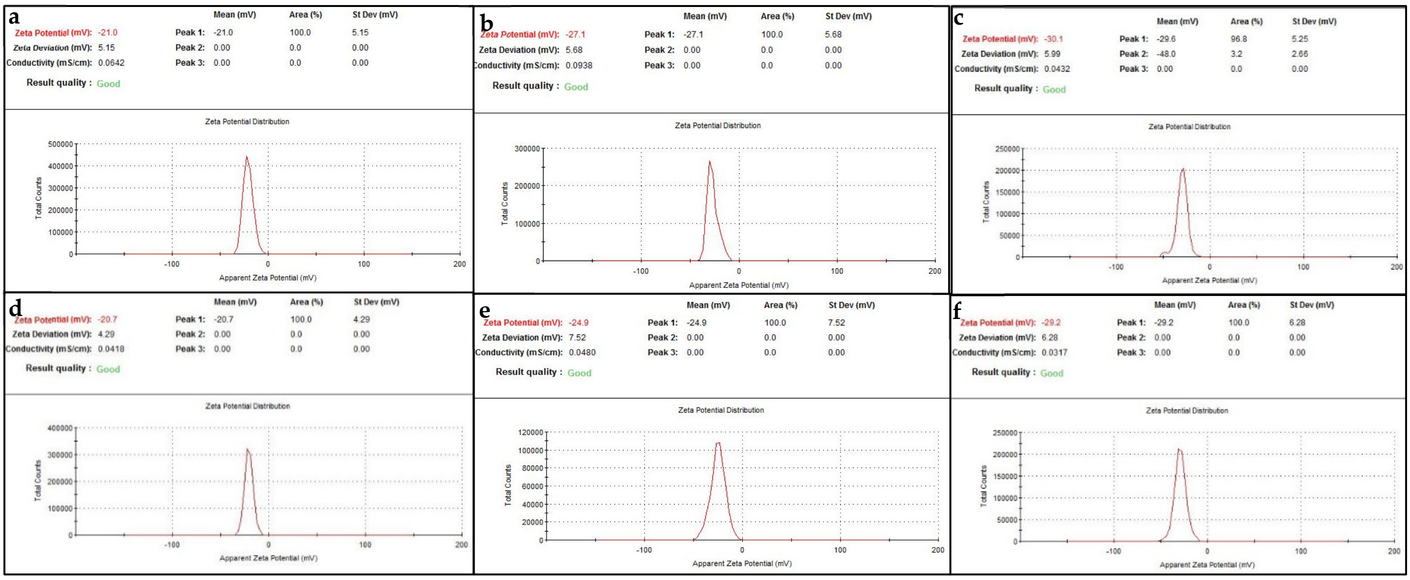

Figure S4. Zeta potential of lyophilized transfersomes colloid**. (a)** T1, (**b)** T2 (**c)** T3 (**d)** T4, (**e**) T5 and (**f)** T6.
